# Investigating the Function of Adult DRG Neuron Axons Using an In Vitro Microfluidic Culture System

**DOI:** 10.3390/mi12111317

**Published:** 2021-10-27

**Authors:** Rahul Atmaramani, Srivennela Veeramachaneni, Liz Valeria Mogas, Pratik Koppikar, Bryan J. Black, Audrey Hammack, Joseph J. Pancrazio, Rafael Granja-Vazquez

**Affiliations:** 1 Center for Advanced Pain Studies, University of Texas at Dallas, Richardson, TX 75080, USA; rahul.armani3@gmail.com (R.A.); Srivi.Veeramachaneni@UTDallas.edu (S.V.); lvm160030@UTDallas.edu (L.V.M.); Bryan_Black@uml.edu (B.J.B.); 2 Department of Bioengineering, University of Texas at Dallas, Richardson, TX 75080, USA; Pratik.Koppikar@UTDallas.edu (P.K.); joseph.pancrazio@utdallas.edu (J.J.P.); 3 Department of Research, University of Texas at Dallas, Richardson, TX 75080, USA; ash107020@utdallas.edu

**Keywords:** nociception, dorsal root ganglia, axonal stimulation, maladaptive plasticity, drug discovery, microfluidics

## Abstract

A critical role of the peripheral axons of nociceptors of the dorsal root ganglion (DRG) is the conduction of all-or-nothing action potentials from peripheral nerve endings to the central nervous system for the perception of noxious stimuli. Plasticity along multiple sites along the pain axis has now been widely implicated in the maladaptive changes that occur in pathological pain states such as neuropathic and inflammatory pain. Notably, increasing evidence suggests that nociceptive axons actively participate through the local expression of ion channels, receptors, and signal transduction molecules through axonal mRNA translation machinery that is independent of the soma component. In this report, we explore the sensitization of sensory neurons through the treatment of compartmentalized axon-like structures spanning microchannels that have been treated with the cytokine IL-6 and, subsequently, capsaicin. These data demonstrate the utility of isolating DRG axon-like structures using microfluidic systems, laying the groundwork for constructing the complex in vitro models of cellular networks that are involved in pain signaling for targeted pharmacological and genetic perturbations.

## 1. Introduction

A critical role of the peripheral axons of nociceptors of the dorsal root ganglion (DRG) is excitation from peripheral nerve endings to the central nervous system for the perception of noxious stimuli [1]. Plasticity along multiple sites along the pain axis has now been widely implicated in the maladaptive changes occurring in pathological pain states such as neuropathic and inflammatory pain [2]. Notably, increasing evidence suggests that nociceptive axons actively participate through the local expression of ion channels, receptors, and signal transduction molecules through axonal mRNA translation machinery [3,4] that is independent of the soma component. Ultimately, the peripheral terminals, not the soma, are exposed to a myriad of inflammatory cytokines and immune-related molecules during inflammation and/or nerve injury and may contribute to the peripheral hyperexcitability of mouse DRG neurons [5]. Therefore, a more detailed understanding of the role of axons that are independent of the soma in nociceptive plasticity is required.

The ability to physically and chemically isolate peripheral axons for genetic and or pharmacological manipulation may elucidate the role of axons in the maladaptive changes occurring in hyperexcitable nociceptors under inflammatory conditions. However, typical in vitro neuronal cultures have no physical and chemical isolation of axonal processes, and principal measurements increasingly rely on that of cell soma responses. However, advances in microfluidic cell culture platforms have overcome these challenges [6,7,8,9]. Microfluidic structures allow the culture of neuronal cell bodies in a distinct compartment, where axon-like structures extend across microchannels interconnecting a separate distal compartment. Through the physical and chemical isolation of the soma and axonal compartments, microfluidic systems allow spatial and temporal control over the axonal microenvironment, allowing separate characterization of cellular components. Prior studies have utilized DRG neurons in microfluidic structures for imaging and biochemical manipulations; however, these studies used neonatal tissue, which complicates the study of NGF-mediated sensitization [10]. The use of adult tissue is a key differentiating component of the present study and one that allows us to build upon our electrophysiological observations and methodology [5] towards constructing complex in vitro models.

Interestingly, a large proportion of inflammatory and immune-related mRNA is encoded exclusively in adult sensory axons in response to injury compared to embryonic axons [3]. This observation highlights the role of local protein synthesis in mature tissue for the establishment and maintenance of chronic and neuropathic pain states and the importance of adult DRG tissue in modelling degeneration or maladaptive changes following injury and/or inflammation.

In the present study, we report an approach for interrogating the adult DRG neurons cultured in a microfluidic culture system. We demonstrate that this approach allows for the physical isolation of adult DRG cells bodies from their axon-like structures in adjacent compartments. Further, we demonstrate the ability of the axon-like structures to respond to a relevant agonist, capsaicin, while observing soma responses through calcium imaging. We also show that DRG cell bodies respond to the administration of an inflammatory cytokine (IL-6) [5] when introduced into the axonal compartment. Our findings suggest that the DRG axon-like structures spanning microchannels in vitro can convey excitation and mediate sensitization.

## 2. Materials and Methods

### 2.1. Fabrication and Assembly of PDMS Microfluidic Chambers

Two-chambered microfluidic devices were fabricated as described previously with minor modifications [11]. Namely, a ~12.8–15 μm thick layer of SU-8 photoresist (MicroChem, Westborough, MA, USA) was patterned on a silicon wafer (University Wafers, Boston, MA, USA) to serve as the template for the connecting microchannels. This layer was formed by spin coating at 500 RPM with a 1000 ramp for 10 s and then at 1000 RPM with a 200 ramp for 35 s followed by baking at 105 °C for 2 min and exposure to ultraviolet (UV) light at 100 mJ/cm^2^. After exposure, the wafer was baked for 1 min at 70 °C followed by an additional 2 min at 105 °C. Then, it was developed twice for 3 min using a SU-8 developer (MicroChem, Westborough, MA, USA). The wafer was baked for an additional 2 min at 110 °C. During the second step, a ~100 μm thick layer of photoresist (SU-8) was deposited by spin coating at 500 RPM with a 100 ramp for 10 s then at 1000 RPM with a 300 ramp for 40 s followed by baking at 70 °C for 5 min and 20 additional minutes at 105 °C. The wafer was aligned and was then exposed to UV light at 320 mJ/cm^2^. The wafer was baked for 1 min at 70 °C followed by an additional 10 min at 105 °C. It was developed twice for 3 min using SU-8 developer, rinsed with 20 mL IPA, and dried under a stream of nitrogen. The PDMS microfluidic devices were fabricated using previously described methods with minor modifications [12]. Namely, an outlet port was punched directly into the proximal and distal chamber to form an “open well” structure using a sterile 6 mm hole punch. The PDMS devices were bonded to glass cover slides via plasma treatment [12].

### 2.2. Isolation and Culture of Adult DRG Neurons

**Figure 1:**
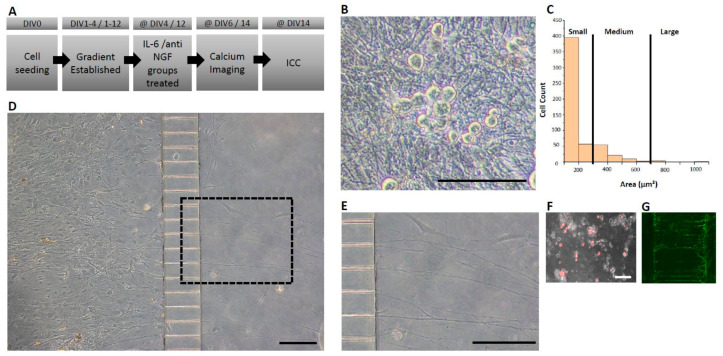
Isolation of axon-like structures in adult mouse DRG neuron cultures in microfluidic-based culture platforms (**A**) Experimental timeline. (**B**) Cell viability observations in phase contrast microscopy depicting healthy neuronal cell bodies. (**C**) Dorsal root ganglia cross-sectional area categorization with the majority of cells being of small-medium range. (**D**) Representative image of a seeded device with the soma (left) and axonal (right) chambers interconnected via microchannels at DIV 6 (**E**) Magnified view of the dashed rectangle from panel D demonstrating isolated axon-like structures crossing microchannels. (**F**) Axon-like structures crossing identified by the fluorescent tracer, Dil, applied to the axonal chamber. Dil is taken up by crossing axons and retrogradely transported to the soma. (**G**) Axon-like structures stained with peripherin, a marker that is preferentially expressed by peripheral sensory neurons. Horizontal scale bars represent 150 μm.

Adult DRG neuron isolation and culture methodology was adapted from Black et al. [5]. All surgical procedures were performed in accordance with University of Texas at Dallas’s Institutional Animal Care and Use Committee. In all experimental cases, male mice (4–6 weeks old; Envigo RMS Inc., Indianapolis, IN, USA) from the Institute for Cancer Research (CD-1) were used. Briefly, after dissociation and isolation of the cell pellet, cells were resuspended in fresh 10–20 μL of complete cell medium. Approximately 4–5 × 10^5^ viable neurons were plated per device in the soma chamber (36 mm^2^ area) of the microfluidic device and were allowed to adhere for 30 min. Following adhesion, complete cell medium, as described in Black et al., 2018 [5], was added to both the soma and axonal chambers. To induce axonal outgrowth across the microchannels and into the axonal chamber, a neurotrophic gradient was established as follows: On day in vitro (DIV) 0 and DIV 1, (Figure 1A) both the axonal and soma chambers were treated with media containing 100 ng/mL of both mouse nerve growth factor (mNGF, Sigma Aldrich, St. Louis, MO, USA) and human glial derived neurotrophic factor (hGDNF, Peprotech, Inc., Rocky Hill, NJ, USA). On DIV 1–3, the media were adjusted to contain 50 ng/mL in the soma chamber and 100 ng/mL in the axonal chamber for both growth factors. From DIV 3 onwards, the media were adjusted to contain 25 ng/mL in the soma chamber and 100 ng/mL in the axonal chamber for both factors and was exchanged every 48 h. Cell viability and axon-like structures were assessed after seeding in a phase contrast microscope (Figure 1B).

In some experiments, cells were also seeded distally (1600 µm) from the entrance to the microchannels (Xona Microfluidics, Inc., Research Triangle Park, NC, USA). All of the devices that were used (custom or Xona) had identical dimensions/design, particularly pertaining to the microchannels which were 150 μm in length, 5 μm in height, 7.5 μm in width, and with an inter-channel spacing of 52.5 μm (Figure 1D). Cultures were maintained in identical growth factor and volume gradient as described above. This set of cultures was maintained over a period of 14 days.

### 2.3. Calcium Imaging

At either DIV 6 (short term cultures) or DIV 14 (long term cultures), complete cell media was removed and media containing the calcium indicator Fluo-4, AM, (5 μM, ThermoFisher Scientific, Waltham, MA, USA) were loaded into both chambers for 30–45 min at 37 °C. Following staining, both chambers were washed once with Hanks’ Balanced Salt Solution (HBSS, Sigma Aldrich, St. Louis, MO, USA) before imaging commenced. Micro-fluidic chambers were directly mounted on the stage of an inverted microscope (Nikon Ti eclipse, Nikon, Tokyo, Japan) equipped with a humidified chamber (OKOLAB USA Inc., San Francisco, CA, USA) maintained at 37 °C and 10% CO_2_. The imaging regime consisted of acquiring fluorescence images (488 nm excitation) every 500 ms for 2 min. Isolated axon-like structures were stimulated with 100 nM capsaicin (Sigma Aldrich, St. Louis, MO, USA) reconstituted in complete growth media, and images were acquired from the soma chamber. To ensure chemical and fluidic isolation during the experiment, the soma chamber was maintained in 100 μL of HBSS, and the axonal chamber was maintained at a 50 μL total volume of HBSS.

### 2.4. IL-6 Sensitization of Axons

For the IL-6 sensitization of the axon-like structures, adult DRG neurons were prepared as described above (Section 2.2). On DIV 3, NGF- and GDNF-supplemented media were removed from both the soma and axonal chambers and were replaced with media containing anti-NGF (1:50, Sigma, etc.). Following 24 h of NGF deprivation, cultures were assigned to two experimental groups: axon compartments treated with 100 ng/mL IL-6 and axon compartments continued in anti-NGF for an additional 48 h (negative control). Similar times were employed for the maintenance and group assignment in the long-term cultured devices where IL-6 was added at 48 h prior to calcium imaging experiments. At either DIV 6 (short term cultures) or DIV 14 (long term cultures), axonal responses to 100 nM capsaicin were tested using calcium imaging, as previously described (Section 2.3).

### 2.5. Immunocytochemistry

Following calcium imaging experiments, cultures in the microfluidic chambers were fixed with 4% paraformaldehyde (Sigma Aldrich, St. Louis, MO, USA) and reconstituted in sterile phosphate-buffered solution (PBS) for 30 min at room temperature (RT). Since fixation was performed without the removal of the PDMS microfluidic structures, differential volumes were maintained in the compartments to ensure the fixation of the axon-like structures in the microfluidic channels. Following fixation, both chambers were washed three times for 5 min each with PBS and were stored in PBS + 0.1% sodium azide at 4 °C for at least 24 h. The next day, cells were permeabilized with Triton X-100 (0.2% in PBS solution) for 30 min at RT and blocked with 10% normal goat serum (NGS) (NS02L, Sigma Aldrich, St. Louis, MO, USA) for 2 h. Primary antibody solution was reconstituted in 10% NGS and was added to both chambers and was placed at 4 °C overnight on a plate rocker. Next, chambers were washed with PBS and were treated with secondary antibodies for 1 h at RT. The following primary antibodies were used: anti mouse βIII-tubulin (1:1000, Sigma Aldrich, St. Louis, MO, USA), rabbit anti-peripherin (1:2000, Sigma Aldrich, St. Louis, MO, USA), guinea pig anti-transient receptor potential vanilloid type 1 (TRPV1, 1:1000, Sigma Aldrich, St. Louis, MO, USA), and NeuN (1:500, Abcam, Cambridge, UK). The secondary antibodies were goat anti-rabbit Alexa 405, goat anti-rabbit Alexa 488, or Alexa goat anti-mouse 555. For the visualization of isolectin B4 (IB4), cells were incubated with 1:500 FITC-conjugated IB4 (Sigma Aldrich, St. Louis, MO, USA).

### 2.6. Image Analysis and Data Processing

After staining, microfluidic structures were imaged directly by means of confocal microscopy (Nikon Ti eclipse, Nikon, Tokyo, Japan). Fluorescence and bright field imaging were carried out using an inverted microscope, and all epifluorescent images were acquired using epifluorescent light sources (Lumnecor, Beaverton, OR, USA). Post processing of the calcium imaging data was carried out by first defining regions of interest (ROI) on the cell bodies with an approximate diameter of 30 μm to limit analysis to small-medium gauge neurons, which are presumptive nociceptors. A positive calcium response was attributed to a neuron when the mean fluorescence intensity was greater than three standard deviations above the mean of the baseline, which was calculated from a 30 s window before capsaicin application. Arbitrary unit (AU) data in long term experiments exhibited a downward slope with corresponding positive deflections from baseline, corresponding to calcium transient responses. Raw data were processed in Origin (OriginLabs) using the Asymmetric Least Squares Smoothing filter to define the baseline in this cohort. Data points that deviated from this baseline as positive peaks were then plotted as the change over time from baseline.

Immunocytochemistry (ICC) imaging was performed from the set of long-term cultured devices at a 10× magnification using an inverted microscope (Nikon Eclipse Ti; Nikon, Tokyo, Japan) and epifluorescent light sources (Lumencor, Beaverton, OR, USA). All images were processed in ImageJ (NIH). To quantify an absolute cell count based on positive stains for NeuN, TRPV1, and IB4 independently, we subtracted the background noise and created a mask by adjusting the color threshold to isolate the cells of interest. If the cells were clustered, the mask was further processed as binary, and the watershed tool was applied to differentiate the cells. Using the “analyze particles” function, we defined a lower bound threshold (400–infinity) and corrected the circularity (0.2–1.0). The same ImageJ protocol was adjusted for TRPV1 channel quantification by changing the upper bound threshold (0–1000) and correcting the circularity (0–1.0). To obtain the co-localization counts for NeuN + TRPV1-positive soma and NeuN + IB4-positive soma, the ImageJ plug-in JACoP was used to generate a masked image of the co-localized regions followed by analyzing the particles to obtain a cell count.

Size-based neuronal DRG analysis: At least three diameters of NeuN positively stained cells were averaged using ImageJ (NIH), and the surface area was calculated for each cell. This method was utilized for each DRG cell due to their spherical shape variability. Cells were then classified based on previous size-based characterizations of DRG cell subtypes [5,13].

### 2.7. Statistical Analysis

A two-sample t-test was performed to compare the means of the two groups to test the hypothesis of there being no difference between the control and treatment groups. For all conditions, a *p* value < 0.05 was considered statistically significant. Unless otherwise noted, all descriptive statistics are represented as the mean ± standard error of the mean (SEM).

## 3. Results

### 3.1. Characterization of Adult DRG Neuron Preparations in Microfluidic Chambers

Microfluidic systems allow temporary fluidic isolation between the soma and axonal chambers, which is established by a volume difference between chambers. The resulting fluidic pressure generated at the microchannels minimizes the diffusion of molecules. This is especially important for the generation of a stable neurotrophic gradient to stimulate axonal growth across microchannels into the axonal chamber. We and others have previously demonstrated a temporary chemotactic field across the microchannels using FITC-conjugated dextran to mimic the diffusion profile of growth factors and/or cytokines [11,14,15]. Our prior work showed no fluorescence intensity in the soma compartment via fluorescence microscopy after the establishment of a gradient for a minimum of 8 h, and this was maintained for at least 24–48 h [11]. We created a neurotrophic gradient to stimulate axonal growth across the microchannels using nerve growth factor (NGF) and glial-derived neurotrophic factor (GDNF). DRG neurons were plated in the soma chamber (Figure 1D), and the axonal compartment was treated with a combination of NGF and GDNF, and crossings were monitored over a 6-day period. Axonal crossings in the distal chamber were identified as early as 24 h and by DIV 6, a large proportion of crossings were visible across all micro-channels (Figure 1D,E). To quantify the percentage of neurons extending axon-like structures, a fluorescent tracer (Dil) (1/200) was added to the axonal chamber for 2 h in the short-term experiments. As a lipophilic membrane stain, Dil was readily incorporated into the membrane of the axon-like structures and retrogradely transported to the cell soma where the stain is accumulated, resulting in a maximized signal. At DIV 6, we found that 80 ± 3 % (*n* = 3 devices, *n* > 100 cells/device) of the cell bodies were positively stained with Dil. Furthermore, approximately 99% of neuronal DRG cells were found to be in the range of small to medium (0–700 μm^2^) based on their cross-sectional areas (Figure 1C).

These data suggest that we were able to create a culture with a high percentage of nociceptive neuronal cells, as described in previous size-based discrimination cell subtype studies. While we observed that 80% of the total cells were positive for the retrograde tracer (Figure 1F), we further corroborated these results by demonstrating that the axonal crossings detected in the distal chamber are positive for peripherin (Figure 1G)—a positive marker for TRPV1-expressing neurons that are C-fiber nociceptors— in preparations of mouse dorsal root ganglia [16].

### 3.2. Characterization of DRG in Microfluidic Devices

**Figure 2:**
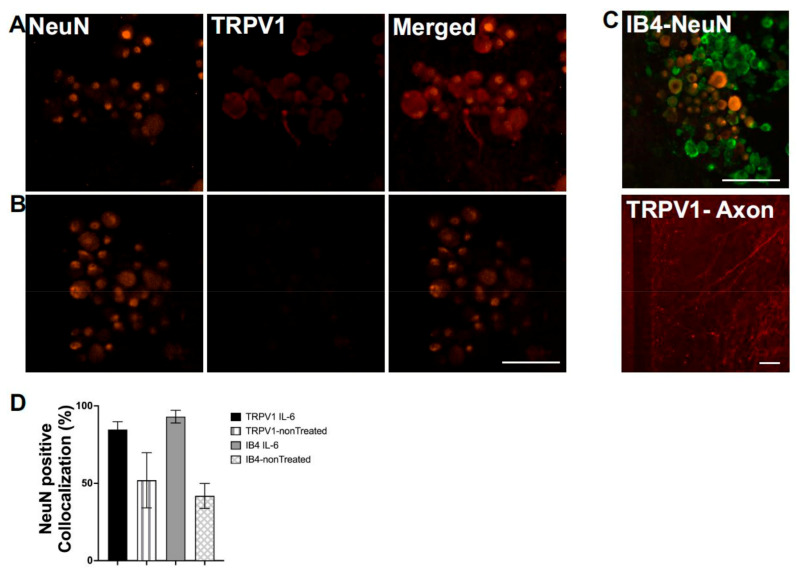
Immunocytochemistry (ICC) profile of cultures in microfluidic devices at day in vitro 14: (**A**,**B**) Representative images of an IL-6-treated culture (**A**) and a vehicle-treated culture (**B**) with NeuN positive cells (orange) that are indicative of neuronal cell bodies. We quantified NeuN co-localization with nociceptive markers TRPV1, which are shown in red, and scale bar represents 100 μm. (**C**) Representative images of IL-6-treated cultures with NeuN colocalized with IB4, which is shown in green. A representative image of an axonal chamber where IL-6-sensitized axon-like structures demonstrated an increased expression of TRPV1, which is shown in red. Horizontal scale bar represents 150 μm. (**D**) Percent colocalization comparing IL-6- vs. vehicle-treated cultures and their expression profile with respect to the neuronal marker NeuN.

Using ICC, we determined the extent of co-localization with nociceptive markers, TRPV1 and IB4, in DRG neurons identified by the neuronal specific marker, NeuN. Figure 2 shows representative ICC images of labeled DRG cell bodies and axon-like structures before and after sensitization with the inflammatory cytokine IL-6, which is implicated in the development of chronic pain [17,18]. Because NGF can also induce inflammatory pathways [19], for these experiments, cultures were treated with anti-NGF anti-bodies at DIV 3 for 24 h. Following NGF deprivation, cultures were divided into two experimental groups: one experimental group received 100 ng/mL of IL-6 in the axonal chamber for 48 h and the control group was continued in anti-NGF antibodies until DIV 6. Under NGF-deprived conditions by DIV 6, DRG neurons, identified as NeuN positive, showed co-localization with TRPV1 (Figure 2A,B), and IB4 (Figure 2C,D) in 52 ± 13% and 42 ± 6% of cells, respectively (*n* = 3 cultures, 552 cells). To examine the effects of axonal sensitization, we characterized the co-expression of NeuN with TRPV1 and IB4 in the soma compartment after IL-6 (100 ng) was administered to the axonal compartment. After 48 h, the co-localization of NeuN and IB4 significantly (*p* < 0.01) rose to 93 ± 4% of cells (*n* = 2 cultures, 295 cells) (Figure 2C,D). The effects of IL-6 were not restricted to cell bodies but also could be observed from the increased labeling of TRPV1 in treated axon-like structures (Figure 2D). Administration of IL-6 significantly (*p* < 0.01) enhanced TRPV1 expression in axon-like structures by 2.7-fold.

### 3.3. Calcium Imaging to Assess Axon Functionality

**Figure 3:**
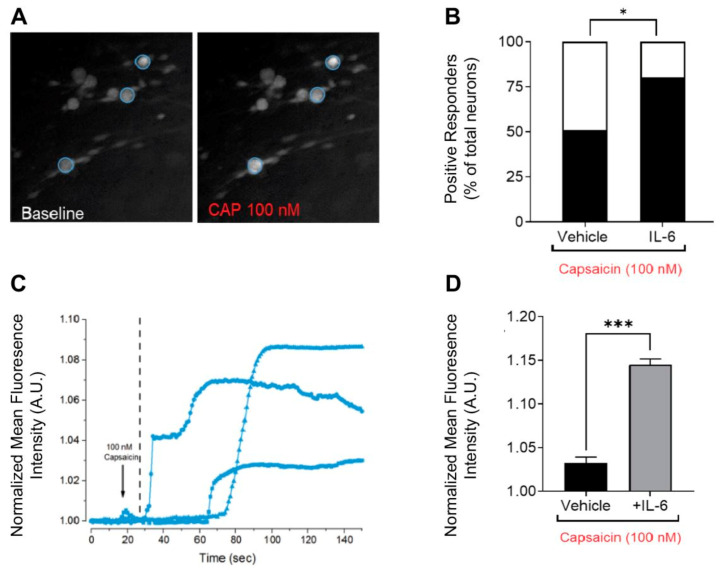
Local treatment of the axonal microenvironment with inflammatory cytokine IL-6 enhances capsaicin-evoked responsiveness. (**A**) Representative soma images in short-term experiments of calcium responses to 100 nM capsaicin applied in the axonal chamber. (**B**) Quantification of the number of positive responders as a proportion of total neurons post-exposure to 100 nM capsaicin in vehicle and IL-6 treated groups, where * denotes test of proportions, *p* = 0.02 (**C**) Representative traces of baseline-normalized fluorescence intensity of calcium influx post-axonal stimulation as a function of time from defined regions of interest. (**D**) Magnitude of the calcium transient recorded post-exposure to 100 nM capsaicin in vehicle- and IL-6-treated groups, where *** denotes two sample unpaired t-test, *p* < 0.0001.

Physically and chemically isolated axon-like structures can be probed by relevant agonists to measure evoked excitability using calcium imaging [20,21,22]. In vivo, action potentials are initiated in the nerve ending of sensory axonal fibers in response to a stimulus in the peripheral receptive field. This signal is transduced and transmitted by axons to the soma for transmission to the central nervous system. Therefore, we postulated that the stimulation of the isolated peripheral axon-like structures with a relevant agonist will result in local axonal depolarization and the propagation of action potentials to the soma, which can be monitored as a calcium influx using a calcium indicator. Exposure of the axonal compartment to 100 nM capsaicin led to an increase in the recorded calcium intensity at the soma (Figure 3A) and was represented by an increase in the calcium signal recorded as a function of time (Figure 3C). Capsaicin-evoked responsiveness significantly increased the magnitude of calcium responses in the cultures treated with IL-6 from 1.03 ± 0.007 to 1.15 ± 0.006 (two sample unpaired t-test, *p* < 0.0001, *n* = 27 and *n* = 26 from *n* = 2 devices) (Figure 3D). Similarly, the number of positive responders to capsaicin were significantly increased from 51% to 80% post 48 h exposure to IL-6 (test of proportions, *p* = 0.02) (Figure 3B).

## 4. Discussion

In the present study, a microfluidic-based system is shown for the culture and study of nociceptive axon-like structures in vitro from adult mouse DRG neurons. The maintenance of differential volumes across microchannels interconnecting the soma and axonal chamber can be exploited for the chemical interrogation of the axonal microenvironment that is independent of the cell body. We demonstrated the physical isolation of axonal processes from >80% of the cell bodies seeded in the soma chamber using a neurotrophic gradient. The axonal processes were isolated from a majority of small-to-medium gauge mouse DRG neurons, which expressed classical markers of nociceptors. We demonstrated that this culture model exhibits soma sensitization after peripheral sensitization via the local administration of IL-6 to the axonal microenvironment. This effect recapitulates the in vivo scenario wherein the peripheral nerve endings of nociceptors are in intimate contact with the inflammatory milieu after nerve injury and/or inflammation. Lastly, using calcium imaging, the soma calcium transients were elicited by axonal exposure to capsaicin [23,24], which is indicative of functional axon-like structures that are capable of conveying excitation.

Chronic pain conditions can cause significant maladaptive changes, rendering peripheral nerve fibers hyperexcitable [25]. Our previous studies have demonstrated an increased protein expression of TRPV1 in the cell bodies of mouse DRG neurons in vitro after 48 h incubation with IL-6 [5]. Therefore, it is highly likely that responses to capsaicin may be mediated by an increased local expression of TRPV1 in peripheral axons, as our observations suggest (Figure 2C).

In addition to immunocytochemistry, other molecular techniques such as flowcytometry and qPCR may provide a detailed assessment of receptor expression. However, such techniques are amenable to cells cultured in suspension or adherent in tissue culture plates. In compartmentalized cultures, the reliable retrieval of both soma and axonal tissue from distinct chambers is non-trivial since axonal projections are observed in the distal chamber at as early as 24 h. Therefore, both soma and axonal receptor expression cannot be assessed in total or uniformly. The in situ staining of intact structures of axon and soma in compartmentalized cultures do not require the removal of tissue for processing and conserve the cellular architecture to interpret results with improved spatial resolution.

Even though the role of axons in response to inflammatory mediators has been addressed in many ways, to the best of our knowledge, this is the first time where this is being addressed in a compartmentalized culture system. While a direct comparison of the effects of IL6 at the soma can be inferred from a conventional culture system, such as from our multi electrode array data [5], there are other potential questions that could be asked and answered in future studies related to axonal translation rates and intercellular signaling in the soma chamber. The understanding of the local effects of IL-6 can be improved via the blockade of retrograde axonal transport. However, the definitive role of IL-6 in maladaptive axonal function changes is controversial, as it has been implicated in mediating both a secondary inflammatory response and axonal sprouting [26]. Elucidating the role of IL-6 in axonal physiology is integral, and future studies will be focused on assessing the use of translational blockers, specifically at the axonal nerve endings, to describe the function and corresponding mechanism of IL-6 in nerve injuries.

Several other inflammatory mediators can be considered in the context of modulating DRG neuronal function. However, in the present study, we focused on IL-6 due to its established role in inflammatory pain. Specifically, our prior reports [5] using disorganized in vitro cultures of DRG neurons have demonstrated that IL-6 treatment may result in an increase in TRPV1 expression, which can be observed at the cell body level. Therefore, the present study investigates, in part, the axonal role in modulating such expression via fluidic and physical isolation to provide a more detailed understanding of DRG pathophysiology under inflammatory conditions. Similar approaches might be tailored to other relevant pro-inflammatory mediators.

While calcium imaging is an important tool in probing neuronal excitability, calcium-based activity is a surrogate measure of neural activity due to slow kinetics relative to changes of the membrane potential. Moreover, since calcium is an important secondary messenger that mediates numerous intracellular processes, the metric is a global response and cannot be readily tied to action potential firing rates or patterns. Long-term monitoring of cellular excitability is not amenable to calcium-based fluorescence dyes due to phototoxicity or photobleaching under long exposure times. Future studies will assess the development of an integrated system, combining substrate integrated microelectrode arrays with PDMS-based microfluidic structures to allow the recording electrodes to interface directly with soma and axonal chambers to allow long term, non-invasive, and high content measures of neuronal excitability. The present findings demonstrate the functionality of DRG axon-like structures crossing through microchannels, laying the groundwork for constructing the more complex cellular networks that are involved in pain signaling.

## Data Availability

Experimental data required to reproduce the findings from this study will be made available to interested investigators upon request.
